# Stabilization of magnetic helix in exchange-coupled thin films

**DOI:** 10.1038/srep16153

**Published:** 2015-11-05

**Authors:** L. V. Dzemiantsova, G. Meier, R. Röhlsberger

**Affiliations:** 1 The Hamburg Centre for Ultrafast Imaging, Luruper Chaussee 149, 22761 Hamburg, Germany; 2 Deutsches Elektronen-Synchrotron, Notkestra*β*e 85, 22607 Hamburg, Germany; 3 Max-Planck Institute for the Structure and Dynamics of Matter, Luruper Chaussee 149, 22761 Hamburg, Germany; 4 Center for Free-Electron Laser Science, Luruper Chaussee 149, 22761 Hamburg, Germany

## Abstract

Based on micromagnetic simulations, we report on a novel magnetic helix in a soft magnetic film that is sandwiched between and exchange-coupled to two hard magnetic layers with different anisotropies. We show that such a confined helix stays stable without the presence of an external magnetic field. The magnetic stability is determined by the energy minimization and is a result of an internal magnetic field created by the exchange interaction. We show that this internal field stores a magnetic energy density of a few kJ/m^3^. We also find that it dramatically modifies ferromagnetic resonances, such that the helix can be used as a ferromagnetic resonance filter and a fast acting attenuator.

Robust and energetically efficient magnetic structures that employ the spin degree of freedom to store and process information are at the heart of modern spin-based technology^1^,^2^,^3^. Many experiments have been performed to investigate the interaction of spins with electrical charges or external magnetic fields, using different device geometries like mechanically or lithographically fabricated point contacts, nanopillars or tunnel junctions^4^. It has recently been shown that the transmission and processing of information without electric currents or external fields can be achieved via the spin degree of freedom subjected to internal interactions such as exchange, Ruderman-Kittel-Kasuya-Yosida (RKKY) or long-range dipolar interactions^5^,^6^. In combination with boundary conditions including magnetic anisotropy, these interactions can topologically stabilize spin configurations like spin helices^5^,^7^ without presence of chiral Dzyaloshinskii-Moriya interaction^8^,^9^,^10^. In the work of E. Y. Vedmedenko *et al.*^5^, it has been pointed out theoretically that nano-sized stable helices can be used for magnetic energy storage. Although a large variety of magnetic devices with desirable parameters can be fabricated down to the sub-nanoscale, the creation of helices with stable magnetic properties can, however, be an experimental challenge.

Here, we propose an approach for creating novel magnetic helices in exchange-coupled thin films. We show that such helices, initially twisted in an external magnetic field, stay stable even without the presence of the field. In contrast to the helimagnetism governed by the RKKY interaction in rare-earth materials^11^,^12^, or by the dipolar interaction in a [Co/Si] × 3 multilayer^13^, the functionality of magnetic helices in this study relies critically on the exchange-coupling mechanism of thin layers consisting of a hard and a soft-magnetic material. As a characteristic property of exchange-coupled layers or exchange spring magnets, the magnetization of the soft-magnetic film is pinned to the hard-magnetic film at the interface as a result of the exchange interaction^14^. With increasing distance from the interface, the exchange coupling becomes weaker and the magnetic moments in the soft layer form a spiral structure under the action of an external field. To stabilize this spin spiral structure, we add on top a magnetic film with an anisotropy value that lies in between those of the hard and the soft material. When the external field is removed, such a trilayer can relax into a new stable configuration where a magnetic helix exists. The magnetic stability is the result of an internal field that is created by exchange interaction and stores magnetic energy. We find that this field dramatically modifies ferromagnetic resonance (FMR) frequencies in the GHz range, compared to the untwisted ferromagnetic state at zero applied magnetic fields.

In experiments, magnetic materials such as FePt in the hard magnetic tetragonal L1_0_ phase and Fe can be used as the bottom hard and mid soft magnetic layers, respectively^15^. The top layer with a coercive field up to 100 mT can be obtained by sputtering Fe via a technique known as oblique incidence deposition (OID) at room temperature^16^,^17^. The magnetic helix can be studied with Mössbauer spectroscopy^18^, nuclear resonant scattering^19^, resonant magnetic x-ray reflectometry^20^ or polarized-neutron reflectometry^21^, which all are capable to characterize vertical spin profiles in multilayers. Using broadband FMR with a vector network analyzer^22^, one can distinguish the helix from the ferromagnetic alignment.

Nanocomposite materials with a stable helical order open broad perspectives for future scientific and technological applications in the field of spin engineering on smallest length scales. Since these structures store magnetic energy, they can serve as energy-storing elements in spin-based nanodevices. The stored energy can be released by switching the magnetization of the top layer with a laser pulse^23^,^24^, a radio-frequency field pulse^25^ or a current pulse^26^,^27^, and transformed into its mechanical, electric or magnetic counterparts. The generation of high-frequency signals without the presence of external fields or electrical currents renders magnetic helices promising candidates for application in field- or charge-free spin transport effects in nanoscale magnetic schemes.

## Results

### Metastable state

**Figure 1:**
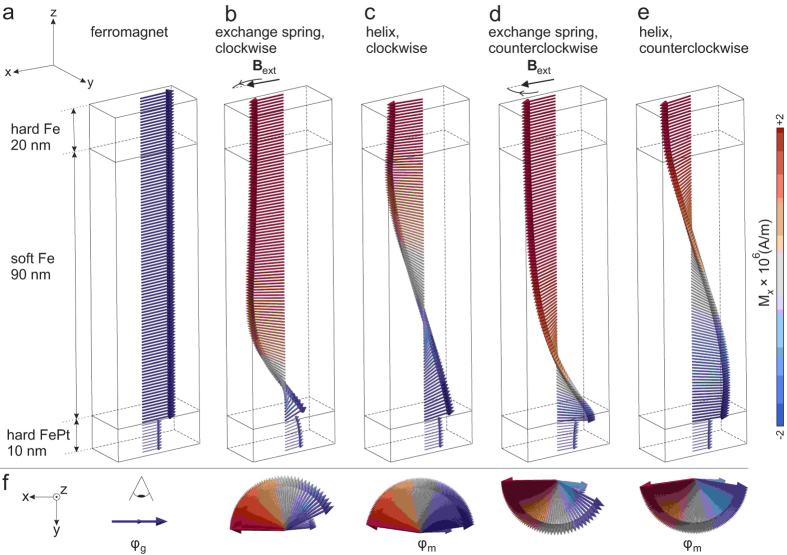
3D representation of micromagnetic simulation data for a hard FePt/soft Fe/hard Fe trilayer with localized magnetic moments (arrows inside slabs) arranged into (**a**) ferromagnet, (**b,d**) exchange spring, (**c,e**) helix. As the initial state, the trilayer shows a ferromagnetic alignment of moments (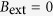). Under the influence of an external magnetic field, 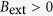, applied to the *whole* volume of the trilayer, the exchange spring can be created. When the external field is removed, the trilayer can relax into the metastable state, where the helix exists. The counterclockwise/clockwise rotation direction of *B*_ext_ defines the clockwise/counterclockwise chirality of the exchange spring, hence the helix. (**f**) Top view of the trilayer. *φ*_g_ (*φ*_m_) is the relative angle between the magnetization directions of the bottom and top hard magnetic layers, when the trilayer is in the ground state (metastable state). The colorscale highlights the *x*-component of the magnetic moments, *M*_*x*_, with respect to the minimum (blue) and the maximum (red) value.

We model the film as a layered structure, where the individual layers differ in thickness *d*, saturation magnetization *M*_*s*_, and magnetic anisotropy *K* with an uniaxial magnetization axis in the *xy*-plane. As shown in Fig. 1(a), the structure consists of three magnetic films: (*i*) a hard magnetic FePt layer, (*ii*) a soft magnetic Fe layer and (*iii*) a hard magnetic Fe layer. In the following, we simply call a soft (hard) magnetic layer a soft (hard) layer. As an initial state, the relative angle between the magnetization directions of the bottom and top hard layers is *φ*_g_ = 0°, i. e. the layer stack exhibits a ferromagnetic alignment of moments (see Fig. 1(a)). The angle *φ*_g_ can be adjusted between 0° and 90°, controlled by the OID technique during preparation^16^,^17^.

**Figure 2:**
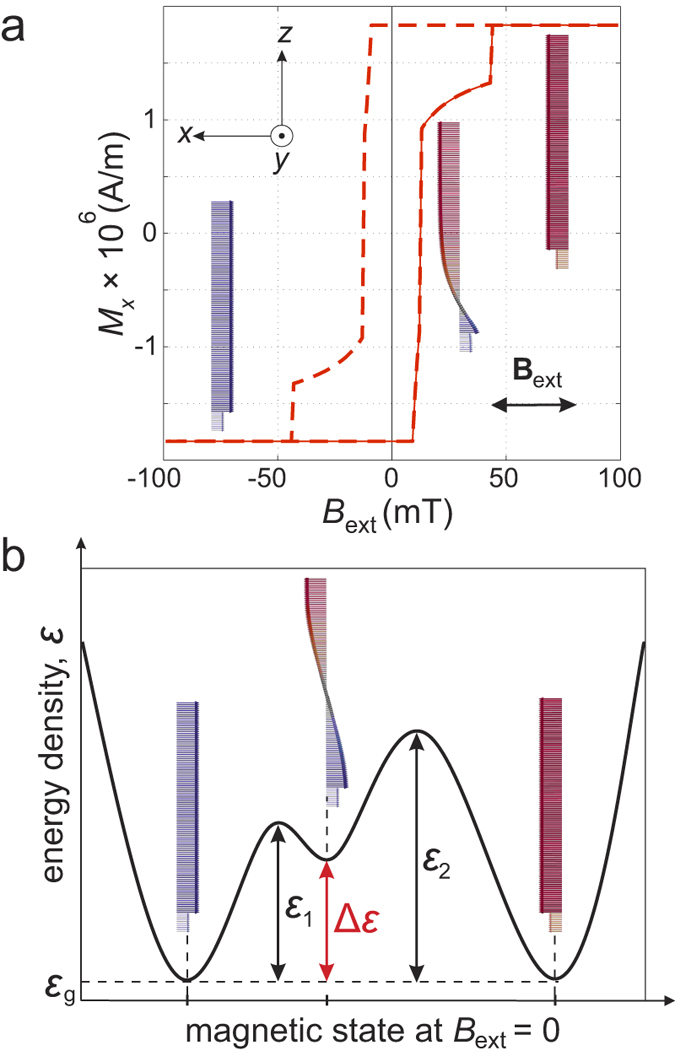
(**a**) Hysteresis loop (dashed) and minor loop (solid) of the trilayer shown in Fig. 1. For the minor loop, the external magnetic field 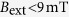 (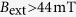) saturates all magnetic moments in the negative (positive) direction. In the field range of 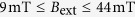, the magnetic moments are arranged into the exchange spring. (**b**) Schematic drawing of the potential energy density of the trilayer versus magnetic state at 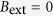. Energy density barriers *ε*_*i*_ (*i* = 1, 2) separate two global minima at the ground state *ε*_*g*_. A local minimum in between corresponds to the metastable state, where the helix exists. Inserts are the representation of micromagnetic simulation data.

To find an equilibrium state other than the initial state, we perform micromagnetic simulations, thereby repeating the following procedure for increasing values of an external magnetic field *B*_ext_ applied to the *whole* volume of the stack: Starting with 1 mT, we rotate *B*_ext_ by 180° relative to the initial direction of the magnetic moments in the hard Fe and relax the trilayer. The field is then set to zero and the structure is again relaxed. The procedure is repeated for 2 mT, 3 mT up to 100 mT. At each step, we observe the magnetic state after the relaxation in the external field and subsequently without it. Under the influence of the clockwise (counterclockwise) rotated external field, the magnetic moments in the Fe films are spatially twisted counterclockwise (clockwise) due to exchange coupling between FePt and Fe through the interface as shown in Fig. 1(b,d). Regardless of the rotation direction, we find that the exchange spring exists at external magnetic fields of 9 mT up to 44 mT (see the hysteresis loop in Fig. 2(a)) for the layer stack mentioned above. With further increase of the field strength, the magnetic moments in the stack completely switch by 180°.

Depending on the energy density introduced by the external magnetic field, *ε*_ext_, the modeled stack can relax into different equilibrium states, when the external field is removed. Figure 2(b) schematically illustrates the energy density landscape of the stack, with two global minima at *ε*_g_ and one local minimum in between, separated by energy density barriers *ε*_i_ (*i* = 1, 2) (see Table S1 in the Supplementary Information). If *ε*_ext_ < *ε*_1_ (*ε*_ext_ > *ε*_2_), the trilayer relaxes into a ground state with a global minimum, where all magnetic moments are aligned parallel (antiparallel) to those in the initial state (Fig. 1(a)). In case of *ε*_1_ ≤ *ε*_ext_ ≤ *ε*_2_, the stack relaxes into the metastable state with the local energy minimum. In this state, the magnetic moments in the soft layer are arranged into a helix. Pinned to the antiparallel magnetic moments of the hard layers at the ends, this helix is stable, i.e. the ground state cannot be reached by continuous deformation^5^,^7^. The helix can show clockwise (Fig. 1(c)) or counterclockwise (Fig. 1(e)) rotation, which depends on the rotation direction of the external magnetic field applied before. We observe the helix after the magnetic moments were twisted in external fields in the range of 13 mT to 38 mT for the layer stack mentioned above.

### Internal magnetic field

**Figure 3:**
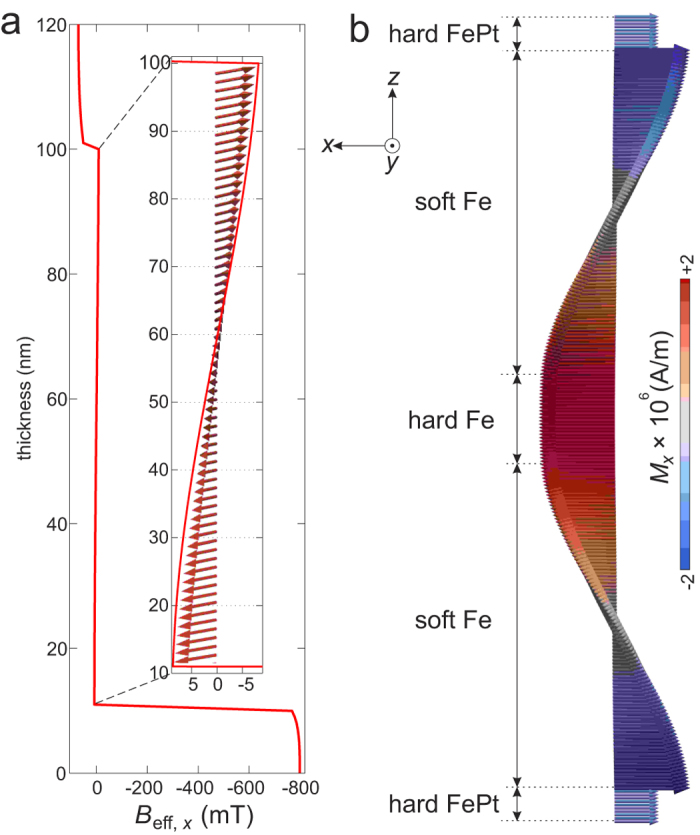
Micromagnetic simulation data: (**a**) the *x*-component of the internal effective magnetic field, *B*_eff,*x*_, as a function of the thickness in the helix shown in Fig. 1(e). The insert is the representation of the internal effective field, *B*_eff_. (**b**) A double-helix constructed from two single helices which are placed up-side-down on top of each other. The colorscale highlights the *x*-component of the magnetic moments, *M*_*x*_, with respect to the minimum (blue) and the maximum (red) value.

The magnetic stability is obtained by energy minimization, which is equivalent to an internal effective magnetic field in the structure. We find that the internal effective field in the helix differs from that in the ferromagnet. In particular, there is the internal effective field in the soft layer in the helix (Fig. 3(a)). To create such an internal field, the following conditions have to be fulfilled: (*i*) An external magnetic field has to be applied to the structure in order to twist the magnetic moments in the Fe layers. (*ii*) Under the influence of the external field, the magnetic moments of the hard FePt film at the bottom have to remain unchanged due to its large anisotropy. (*iii*) When the external field is removed, the magnetic moments of the hard Fe film on top do not have to reverse: its anisotropy has to be large compared to the exchange interaction that tends to unwind the exchange spring and is weaker with distance from the hard FePt magnet underneath. As it can be seen in Fig. 3(a), the internal field in the soft layer forms a spiral, and is held by the magnetic anisotropy in the hard layers at the ends. The magnitude of this field is about 9 mT that is a factor of 90 and 8 smaller than the magnetic anisotropy field in the hard layers at the bottom and on top, respectively. We also find that the internal effective field in the helix stores magnetic energy density Δ*ε* (see Fig. 2(b)). The exchange energy is the main contribution to Δ*ε*, meaning that the internal field in the soft layer is mainly the exchange field.

### Energy storage

Regardless of the chirality, the trilayer in Fig. 1 stores an energy density of Δ*ε* = 7.7 kJ/m^3^. The thermal stability of such an energy storage is limited by the volume of the stack, *V*, and depends on the energy barrier 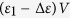 separating the stable states, which should be at least 40 *k*_B_*T*^2^. We find that the smallest volume of the stack that is stable at room temperature is *V*_min_ = 40*k*_B_*T*/(*ε*_1_ − Δ*ε*) ≈ 4 × 10^4^ nm^3^, where *ε*_1_ = 12 kJ/m^3^. This means that the stack with the thickness of 120 nm can be about 6 × 6 nm^2^ in *xy* dimension, which indicates the potential for an energy storage element of an extremely small size.

We consider two main possibilities to increase the stored energy: (*i*) by increasing the number of helices. In this case, trilayers can be duplicated and placed up-side-down on top of each other, as shown in Fig. 3(b). (*ii*) By increasing the twist of the helix, *φ*_m_. As mentioned above, the relative angle between the magnetization direction of the bottom and top hard magnetic layers, *φ*_g_, can be adjusted between 0° and 90°, controlled by the OID during preparation^16^,^17^. If *φ*_g_ > 0°, the magnetic structure in the ground state is a helix with a twist of *φ*_m_ = *φ*_g_. To obtain an even larger twist, an external magnetic field is required that rotates magnetic moments of the top layer clockwise/counterclockwise, while those of the bottom layer remain unchanged. For the trilayer with *φ*_g_ > 0°, the degeneracy between the two chiralities is removed. The helix with the clockwise rotation shows larger twist and stores more energy than those with the counterclockwise rotation (see Table S1 in Supplementary Information). Since we apply the external magnetic field to the whole volume of the stack, the largest twist in the helix is limited to *φ*_m_ = 270°. The energy density stored in such a helix is Δ*ε* = 15.2 kJ/m^3^.

### Ferromagnetic resonance

**Figure 4:**
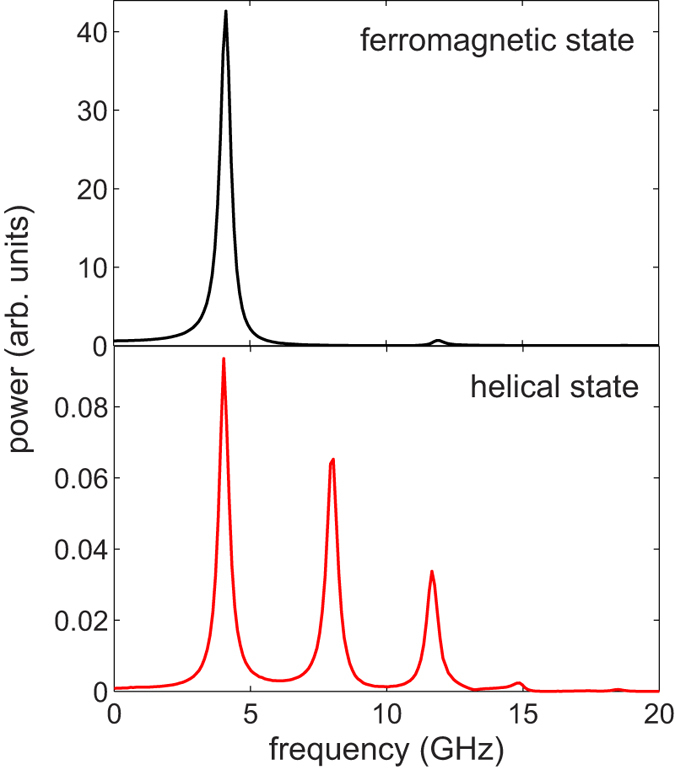
Simulated FMR spectra for the ferromagnetic and the helical state shown in Fig. 1(a,c), respectively. Due to the presence of the internal effective magnetic field, the spectrum in the helix is dramatically modified compared to the ferromagnetic case. In all cases, 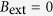.

The energy stored in the internal field has also strong implications on the ferromagnetic resonance frequency, which is crucial for transmitting and processing information in thin multilayers via spin waves. Figure 4 shows the simulated FMR spectra for the ferromagnetic (Fig. 1(a)) and the helical state (Fig. 1(c)). In case of the ferromagnetic state, the spectrum shows only one resonant peak at *f* = 4 GHz that corresponds to a spatially uniform mode^28^. The power of this resonance is, however, strongly reduced, when the structure is in the helical state. The spectrum for the helix also shows resonances at higher frequencies, *f* = 8 GHz and *f* = 12 GHz. We conclude that the presence of the internal magnetic field in the helix causes the dramatic modification of the ferromagnetic resonance spectrum. In order to adjust the resonance frequency in the helix, one can modify the relative angle between the magnetization direction of the bottom and top hard magnetic layers, from 0° and 90°, controlled by the OID during preparation (see Supplementary Information). Also, one can change the film thickness or use different magnetic materials to make a trilayer with a stable helix.

## Conclusion

We have demonstrated theoretically a structure with tunable magnetic properties. The structure consists of exchange-coupled thin films and can be reversibly brought into one of two magnetically stable states: ferromagnetic ground or helical metastable. The helical state originates from the exchange field in the soft layer, held by the magnetic anisotropy of the hard layers at the ends. This differs from chiral magnets, where the helical ground state is caused by the Dzyaloshinskii-Moriya interaction and stabilized by the strain-induced magnetic anisotropy by the substrate^29^,^30^. Contrary to the exchange-coupled films, the ferromagnetic state in chiral magnets can be only observed when an external magnetic field is applied. By switching between ferromagnetic and helical states, the exchange-coupled thin films can be used as a device to store a magnetic energy density of a few kJ/m^3^. Alternatively, it can be used as a filter and a fast acting attenuator of ferromagnetic resonances in the GHz range. The lateral size of such a device can be reduced down to a few nanometers. The proposed nanocomposite with a stable helical state provides a new route to store, transmit and process information without the presence of external magnetic fields or currents.

## Method

Our approach is described within a micromagnetic model utilizing the MicroMagnum code that computes the Landau-Lifshitz-Gilbert (LLG) equation on a grid of coordinates within the sample volume^31^. The effective magnetic energy that enters the LLG equation via the effective field is the sum of exchange energy, demagnetization energy, anisotropy energy and external magnetic field energy. In the present study, we consider a thin film discretized into 2 × 1 × 120 cells in the *x* × *y* × *z* direction, respectively, with 200 repetitions in *xy* dimension. The different choice of the discretization, however, does not change the obtained results. The cell size is 5 × 5 × 1 nm^3^. The film consists of three magnetic layers with the following parameters: an FePt layer (*d*_1_ = 10 nm, *M*_s1_ = 1.1 × 10^6^ A/m, 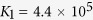 J/m^3^), an Fe layer (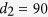 nm, 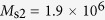 A/m, 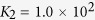 J/m^3^), and an Fe layer (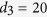 nm, 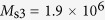 A/m, 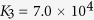 J/m^3^). The exchange constants of all materials are chosen to be 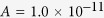 J/m, according to ref. 15. The Gilbert damping constants for FePt and both Fe layers are 0.02 and 0.01, respectively. Magnetization dynamics are studied after a Gaussian pulse of 1 ps duration and 1 mT amplitude is applied in *z*-direction. The external magnetic field is set to zero. The precessional motion is simulated for 3 ns with a time resolution of 25 ps. To obtain a frequency spectrum of the oscillations, we analyze the dynamic response by a Fast Fourier Transformation (FFT) of the *z*-component of the magnetization averaged over all layers.

## Additional Information

**How to cite this article**: Dzemiantsova, L.V. *et al.* Stabilization of magnetic helix in exchange-coupled thin films. *Sci. Rep.* **5**, 16153; doi: 10.1038/srep16153 (2015).

## Supplementary Material

Supplementary Information
