# Supplementary material for: Stabilization of magnetic helix in exchange-coupled thin films
Source: Sci Rep. 2015 Nov 5;5:16153. doi: 10.1038/srep16153 (PMC4633621; doi:10.1038/srep16153)
Supplement: Supplementary Information [file srep16153-s1.pdf]

SUPPLEMENTARY INFORMATION

Stabilization of magnetic helix in exchange-coupled thin films

L. V. Dzemiantsova, G. Meier and R. Röhlsberger

ground state		metastable state, counterclockwise				metastable state, clockwise			
φ_g (°)	ε_g(kJ/m^3)	φ_m (°)	ε_1(kJ/m^3)	ε_2(kJ/m^3)	$\Delta\varepsilon$(kJ/m^3)	φ_m (°)	ε_1(kJ/m^3)	ε_2(kJ/m^3)	$\Delta\varepsilon$ (kJ/m^3)
0	0.0	-180	12.0	20.0	7.7	180	12.0	20.0	7.7
10	0.0	-170	10.5	18.7	6.8	190	12.3	21.5	8.5
30	0.2	-150	8.2	15.2	5.1	210	12.7	21.6	10.2
45	0.5	-135	6.6	12.9	3.8	225	14.8	21.9	11.4
60	0.9	-120	5.5	12.2	2.5	240	17.0	21.9	12.7
90	1.9	-90	5.4	5.4	0.0	270	21.5	24.4	15.2

Table S1 Micromagnetic simulation results for the trilayer shown in Fig. 1 in different ground and metastable states. φ_g (φ_m) is the relative angle between the magnetization directions of the bottom and top hard magnetic layers, when the trilayer is in the ground state (metastable state). ε_g is the energy density of the initial state. ε_1 *and* ε_2 are energy density barries. $\Delta\varepsilon$ is the energy density stored in the helix. The sign " - " indicates the counterclockwise rotation of the helix.
